# 

**Published:** 1891-01-03

**Authors:** 


					" Undoubtedly many persons have been starred to death through inexperienced medioal men SOLD BY
Land nurses placing an undue nutritious value on suoh preparations asExtraot of Meat, home- ...rljmT(, nr,.?r.? .
made beef-tea, fro., whereas had JOHNSTON'S BoVBIL been used In their stead, CHEMISTS, 6R00ERS. &C,
tassa ww w. ctsss-sssk TUET,tl?iu?eK?n?
the disease under GpsJ /Ms\^ which they were THE UNITED KINGDOM.
suffering. I have no m\ Ml JM la^\ hesitation to advise you
-J your patients,to your convalescents, to those of SMx c^> - your clients who have mental exertion*, to use Johnston's
WoL, in g concentrated form, contains a substantial '  " tonic and a palatable food."?J. M, BEAUSOLIEL,;M.D.
No. 223. Vol IX.] Saturday,
January *3rd,?l 891. ~

				

